# Innovative Dental Stem Cell-Based Research Approaches: The Future of Dentistry

**DOI:** 10.1155/2016/7231038

**Published:** 2016-08-28

**Authors:** Shayee Miran, Thimios A. Mitsiadis, Pierfrancesco Pagella

**Affiliations:** Orofacial Development and Regeneration, Institute of Oral Biology, Centre for Dental Medicine, University of Zurich, 8032 Zurich, Switzerland

## Abstract

Over the past decade, the dental field has benefited from recent findings in stem cell biology and tissue engineering that led to the elaboration of novel ideas and concepts for the regeneration of dental tissues or entire new teeth. In particular, stem cell-based regenerative approaches are extremely promising since they aim at the full restoration of lost or damaged tissues, ensuring thus their functionality. These therapeutic approaches are already applied with success in clinics for the regeneration of other organs and consist of manipulation of stem cells and their administration to patients. Stem cells have the potential to self-renew and to give rise to a variety of cell types that ensure tissue repair and regeneration throughout life. During the last decades, several adult stem cell populations have been isolated from dental and periodontal tissues, characterized, and tested for their potential applications in regenerative dentistry. Here we briefly present the various stem cell-based treatment approaches and strategies that could be translated in dental practice and revolutionize dentistry.

## 1. Introduction

Repair of dental pulp and periodontium remains an immense clinical challenge since human teeth have a very limited capacity to regenerate [1]. Current therapeutic interventions in dentistry are based on sophisticated biomaterials and implants with still questionable efficacy and durability [2–5]. Moreover, these therapies do not always allow the appropriate physiological function of the tooth organ. Therefore, there is an enormous unmet need for innovative methods enabling a balance between new dental tissue formation and unaltered physiological functions of the tooth organ [6, 7].

In order to reconstruct such natural structures we need to have a deep knowledge of the cellular and molecular events linked to odontogenesis. Classical experiments have shown that teeth form as a result of sequential and reciprocal interactions between cells of the oral epithelium and neural crest-derived mesenchyme [8]. Epithelial cells differentiate into enamel-forming ameloblasts, while mesenchymal cells give rise to the dental pulp, the dentin-secreting odontoblasts, and the periodontal ligament cells that anchor the tooth to the surrounding alveolar bone [8, 9]. The dental pulp forms a connective tissue that conveys vascularization and innervation and hosts stem cells that are capable of regenerating this tissue, as well as the dentin [1]. Sensory nerves from the trigeminal ganglia (TG) and sympathetic nerves from the superior cervical ganglia innervate the adult teeth [10–12]. During odontogenesis, nerve fibers emanate from the TG project towards the developing tooth germs and progressively surround them without entering the dental pulp. The first axons penetrate the dental pulp when odontoblasts differentiate and enamel deposition starts. Dental pulp innervation is completed soon after tooth eruption into the oral cavity [10], a process that is concomitant with root growth, cementum matrix deposition, and periodontium formation [7].

A common issue in dental practice is the infection and the subsequent extirpation of the dental pulp. Since structures and cells within the dental pulp provide trophic support, sensation, and defense against the various pathogens, devitalized teeth (e.g., after classical endodontic therapy) are subject to severe complications that cause tooth fragility and fracture [13]. It becomes thus evident that the maintenance of dental pulp vitality is of prime importance and therefore new regenerative approaches have started to be experimented in endodontic clinics in the last few years [14]. These new regenerative techniques could also apply in other dental disciplines, where current treatment options are based on a constantly increasing number of dental biomaterials and implants [1–4]. For example, tooth loss caused after severe traumatic injuries, periodontitis (i.e., a severe inflammation of the periodontium), advanced carious lesions, age-related alternations, or cancer [6, 7, 15–19] could be resolved by whole new tooth regeneration.

## 2. Stem Cell-Based Regenerative Dentistry

Regeneration involves the replacement of a pathologic tissue, as well as the reconstruction of a missing or lost tissue, by a new one that can ensure its biological function [20]. Regenerative approaches are based on the potential of human stem cells to self-renew and give rise to a diversity of cell types under appropriate conditions [21]. Throughout life, adult stem cells support tissue homeostasis and repair upon injury [6, 22, 23]. Therefore, adult stem cells from different tissues have been isolated, characterized, and tested for their potential applications in regenerative medicine [20]. In particular, mesenchymal stem cells (MSCs) have been the subject of intense investigation due to their accessibility and their potential to differentiate towards the chondrogenic, osteogenic, adipogenic, myogenic, and neurogenic lineages [24].

**Figure 1:**
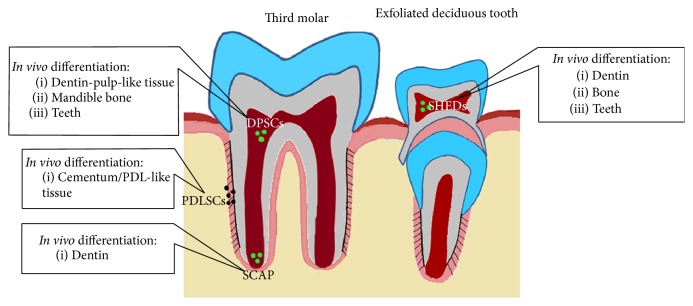
Schematic representation of the main stem cell sources in the human teeth and their differentiation potential* in vivo*. DPSCs: dental pulp stem cells, SHEDs: stem cells from human exfoliated deciduous teeth, PDLSCs: periodontal ligament stem cells, and SCAP: stem cells from the apical part of the papilla.

Different stem cell populations have been isolated from human adult teeth (Figure 1). The dental pulp of third molars is the most common source of dental mesenchymal stem cells, the so called dental pulp stem cells (DPSCs) [25]. Human DPSCs are able to differentiate into odontoblasts, osteoblasts, adipocytes, chondrocytes, and other cell types both* in vitro* and* in vivo* [26–28]. Other populations of dental MSCs (i.e., SHEDs) have been isolated from dental pulp of exfoliated deciduous teeth and from the apical part of the papilla (i.e., SCAPs), similar to the DPSCs differentiation potential [29–31]. Dental MSCs have been also isolated from the periodontal ligament (i.e., periodontal ligament stem cells, PDLSCs) that have the capacity to give rise to cementum/PDL-like tissue* in vivo* [32, 33].

The behavior and properties of stem cells are strongly influenced by the surrounding environment, the so-called stem cell niche [24, 34, 35]. This specific microenvironment is a combination of cells, extracellular matrix, and growth factors that is under the influence by mechanical and chemical stresses [24]. Niches maintain and regulate the balance between stem cell self-renewal and differentiation [35]. In this context, considerable effort is produced to understand how the different components of the niches regulate stem cell function. Among these actors, innervation could play an important role in the fate and function of stem cells [36–42], thus affecting regenerative events. For example, recent studies have shown that parasympathetic nerves regulate progenitor cells and are necessary for the development and regeneration of the salivary glands [37, 39]. Similarly, it has been demonstrated that innervation is essential for the development and maintenance of taste buds [43–46]. Although the role of innervation in the initiation, development, and regeneration of teeth is still highly controversial [34, 47–49], it has been recently shown that sensory nerves regulate MSCs in mouse incisors [42]. Therefore, it is important to better study the role of innervation in tooth regeneration and most particularly its effect in dental stem cell populations. At the same time, it is of equal importance to ensure proper reinnervation of the regenerated dental tissues. Vascularization represents another key aspect of tooth regeneration. The dental pulp is richly vascularized, and teeth depend on blood supply for nutrients and oxygen transport. In addition, blood vessels allow the transport of systemic signals and the recruitment of inflammatory and other circulatory cells into the niches that can strongly affect stem cell function [35]. Blood vessels within the pulp are associated with the so-called perivascular stem cell niches. These niches host MSCs that contribute to the homeostasis and the regeneration of the dental pulp [42, 50]. The growth of blood vessels and nerves often proceeds in parallel, sharing the same paths through tissues [51]. Teeth are peculiar organs in the sense that innervation and vascularization display extremely divergent timing. While blood vessels are found in the pulp of tooth germs during early developmental stages, innervation is established only at late stages of odontogenesis [6, 52]. As both innervation and vascularization are fundamental for tooth physiology and pathology, the identification of the mechanisms that regulate these two processes will be fundamental in the light of partial (e.g., pulp regeneration) or full tooth regeneration.

**Figure 2:**
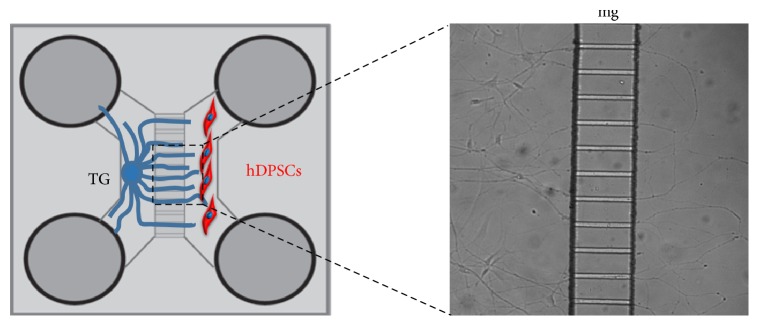
Schematic representation of coculture between trigeminal ganglia (TG) and human dental pulp stem cells (hDPSCs) through microfluidic devices. In this system, both cell types/tissues are cultured in optimal conditions, while allowing the growth of trigeminal ganglia axons through microgrooves (mg) to innervate the stem cells.

Several* in vivo* and* in vitro *studies have provided important information about the patterns of innervation of dental tissues in different animal models as well as the main molecules involved in nerve trophic support, repulsion, and attraction [10, 53–56]. To date, little information is available concerning the effects of innervation on human dental stem cells [57]. Cocultures have been used and constitute a valuable method to investigate and manipulate the interactions between nerve fibers and target tissues of human origin in a controlled and isolated environment (Figure 2) [57, 58]. The use of these and other state-of-the-art techniques, in combination with* in vivo *approaches in animal models, will allow gaining fundamental knowledge regarding the role of innervation in stem cell behavior and the mechanisms that drive tooth reinnervation [6]. To date, only very few studies investigating the possibility of restoring innervation in damaged teeth have been conducted [59].

Pulp revascularization is currently used as a method for its regeneration in not yet fully developed teeth [59]. In this approach, pulp revascularization is induced by filling the empty root canal with a blood clot. Although already in the clinical practice, this method is characterized by low efficiency and by ectopic mineralization within the pulp [59]. Moreover, a major issue that accompanies these revascularization approaches is the connection with the circulation system [59]. Tissue engineering approaches that exploit combinations of functionalized scaffolds and dental stem cells are providing promising results, ensuring variable degrees of pulp regeneration and vascularization* in vivo *in animal models [60–62]. In these studies, good vascularization and connection with the circulatory system were observed, thus suggesting that such approaches could ensure proper trophic support to the regenerated dental tissues [60, 61].

## 3. Clinical Implications: How the Knowledge Obtained from These Approaches Could Be Used

**Figure 3:**
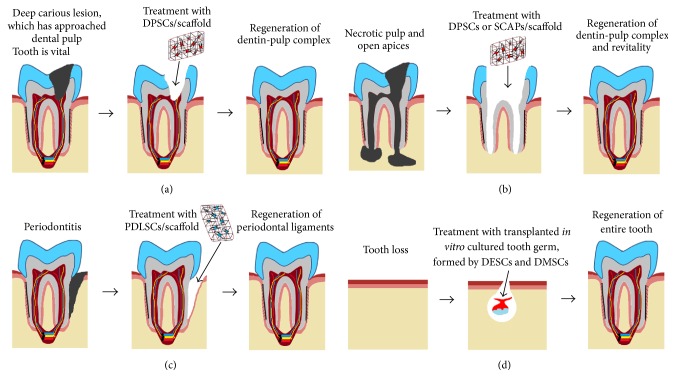
Schematic representation of different stem cell-based approaches for partial or whole tooth regeneration after pathologies and tooth loss using transplantation of tooth stem cells combined with scaffolds. The various dental pathologies: (a) carious lesion, (b) necrotic pulp, (c) periodontitis, and (d) tooth loss. DPSCs, dental pulp stem cells; PDLSCs, periodontal ligament stem cells; SCAP, stem cells from the apical part of the papilla; DESCs, dental epithelial stem cells; DMSCs, dental mesenchymal stem cells.

Stem cell-based therapies are very promising long-term alterative in dentistry since they could offer full restoration of dental tissues keeping thus the structural integrity, physiology, and function of the intact teeth.* In vivo* studies in animals have demonstrated the potential of different tooth stem cells populations for the regeneration of specific dental tissues, such as dentin, pulp, periodontium, or even the entire tooth organ [59, 60, 63–66] (Figure 3). These regenerative approaches that have been successfully tested in animal models could be also applied to humans. It is obvious that these treatments will necessitate a sufficient number of specific stem cell populations that will be transplanted to damaged and pathological dental sites, alone or together with scaffolds.

It is worth noting that stem cell-based approaches have already started to be applied with success in other medical disciplines [67–70]. In dentistry, several specialties took advantage of the recent progress in the fields of stem cell biology and tissue engineering and developed innovative strategies for restoring the full function and physiology of specific dental tissues. For example, regenerative endodontics focuses on reestablishment of dental pulp vitality and new dentin formation using DPSCs/SCAPs combined with scaffolds loaded with bioactive molecules [26, 71–75]. These new procedures allow the transplanted stem cells to differentiate into pulp fibroblasts and odontoblasts and progressively fill the empty pulp chamber after pulpotomy or pulpectomy, thus allowing root growth in not yet fully developed teeth [63, 71, 76–78] (Figure 3). Several clinical attempts based on the bleeding technique and focused in pulp regeneration have been successfully applied in immature teeth with pulp necrosis and mature teeth with apical lesions [71, 78–80]. In this procedure, the blood clot acts as a scaffold that delivers stem cells into the empty root canal.

Although the use of stem cell-based techniques has started to be applied in endodontic clinics, these approaches are still at the animal experimental level for other dental specialties such as in periodontology. For example, while the potential of human PDLSCs or other stem cell populations for periodontal tissue regeneration has been evidenced in animal models [81–84], there are not equivalent trials yet in clinics (Figure 3).

Similarly, although attempts for the regeneration of entire brand new teeth have been successfully performed the last few years in small animal models [85, 86] dental implants still monopolize and offer the therapeutic solution after tooth loss in clinics. However, it is obvious that the ideal therapy after tooth loss would be the regeneration of an entire tooth, and therefore a bigger effort should be produced towards these revolutionary stem cell-based approaches. These novel techniques tested in mice have shown that dental mesenchymal and epithelial stem cells combined with collagen drops or scaffolds* in vitro* allows the formation of tooth germs that thereafter could be transplanted into the alveolar bone, where the tooth germs will develop, erupt, and finally become entire functional teeth [85, 86] (Figure 3). The application of this technique in humans has some limitations. The biggest challenge is the time needed for human tooth regeneration, since the whole process of odontogenesis in humans takes more than 7 years [1, 17]. This may represent a discouragement for patients looking for a quick replacement of the missing teeth.

## 4. Conclusion

Stem cell-based therapies represent the most promising alternative for successful regeneration of damaged or pathological dental tissues or even the entire tooth following tooth loss. It is therefore fundamental to understand the exact mechanisms underlying the potential of the various dental stem cell populations as well as their behavior after transplantation in ectopic sites. Innervation and vascularization play fundamental roles in the regulation of stem cell niches homeostasis, thus affecting the fate and behavior of stem cells [34, 87]. Therefore, it would be of great interest to further investigate the role of innervation in these processes. Stem cell-based approaches are only starting to emerge in dentistry, and huge challenges and problems such the enamel and entire tooth regeneration should be overcome. The majority of the experimental attempts mentioned above were exclusively performed in small animal models and thus they cannot be directly translated into the clinics yet. Nevertheless, stem cell-based regenerative approaches are the future of dentistry that will benefit millions of patients worldwide.
